# Resolving the abundance and air-sea fluxes of airborne microorganisms in the North Atlantic Ocean

**DOI:** 10.3389/fmicb.2014.00557

**Published:** 2014-10-31

**Authors:** Eva Mayol, María A. Jiménez, Gerhard J. Herndl, Carlos M. Duarte, Jesús M. Arrieta

**Affiliations:** ^1^Department of Global Change Research, Institut Mediterrani d'Estudis Avançats, Consejo Superior de Investigaciones Científicas - Universitat de les Illes BalearsMallorca, Spain; ^2^Department of Limnology and Bio-Oceanography, Center of Ecology, University of ViennaVienna, Austria; ^3^The UWA Oceans Institute, The University of Western AustraliaCrawley, WA, Australia

**Keywords:** airborne microorganisms, microbial dispersal, air-sea exchange, bioaerosols, Atlantic Ocean

## Abstract

Airborne transport of microbes may play a central role in microbial dispersal, the maintenance of diversity in aquatic systems and in meteorological processes such as cloud formation. Yet, there is almost no information about the abundance and fate of microbes over the oceans, which cover >70% of the Earth's surface and are the likely source and final destination of a large fraction of airborne microbes. We measured the abundance of microbes in the lower atmosphere over a transect covering 17**°** of latitude in the North Atlantic Ocean and derived estimates of air-sea exchange of microorganisms from meteorological data. The estimated load of microorganisms in the atmospheric boundary layer ranged between 6 × 10^4^ and 1.6 × 10^7^ microbes per m^2^ of ocean, indicating a very dynamic air-sea exchange with millions of microbes leaving and entering the ocean per m^2^ every day. Our results show that about 10% of the microbes detected in the boundary layer were still airborne 4 days later and that they could travel up to 11,000 km before they entered the ocean again. The size of the microbial pool hovering over the North Atlantic indicates that it could play a central role in the maintenance of microbial diversity in the surface ocean and contribute significantly to atmospheric processes.

## Introduction

Airborne microbes are ubiquitous in the atmosphere (Després et al., 2012) and are thought to play important roles in meteorological processes such as the formation of clouds and snow (Christner et al., 2008; Morris et al., 2014), the long-range dispersal of pathogens (Polymenakou et al., 2007) and the maintenance of the diversity in aquatic systems (Hervàs et al., 2009). Global emissions of bacteria have been estimated, based on data from terrestrial environments, to range between 40 and 1800 Gg a^−1^ dry weight (Burrows et al., 2009a). This estimate is poorly constrained due to the paucity of estimates of the abundance of airborne microbes (Burrows et al., 2009a), particularly for oceanic locations, where the few estimates available correspond to coastal locations (Burrows et al., 2009b). The airborne microbial community over the open ocean, comprising the largest share of Earth's surface, has thus far been neglected. Moreover, a large fraction of the available data refer to cultivable bacteria (Burrows et al., 2009b), which yield very low estimates (<1%) of total microbial abundance and diversity (Amann et al., 1995). More recent studies have incorporated direct counting by microscopy or quantitative PCR (Cho and Hwang, 2011; Smith et al., 2012; DeLeon-Rodriguez et al., 2013), yielding more accurate, higher estimates of the abundance of airborne microbes. Flux measurements are even more scarce and mostly limited to land and coastal locations since direct measurements are virtually impossible on the open sea (Petelski and Piskozub, 2006).

Hence, our knowledge on airborne microbes over the oceans is very limited compared to that on microbes inhabiting aquatic and terrestrial environments. Addressing this gap requires that estimates of the abundance of airborne microbes need to be extended to include the atmospheric boundary layer over the ocean, and that the dynamics of the airborne bacteria, in terms of the atmospheric transport of microbes and the exchange of microbes between the atmosphere and the ocean, be quantified to assess the impact of airborne microbes on climate and atmospheric processes (Morris et al., 2014).

In this study we establish an approach for the quantitative assessment of the abundance of microorganisms in the atmosphere over the open ocean using commercially available equipment. Air-sea exchange of microbes and the potential for dispersal were then estimated using available measurements and different parameterizations resulting in the first quantitative estimates of abundance and air-sea exchange of microorganisms for the North Atlantic Ocean.

## Materials and methods

### Sampling and microbial abundance

**Figure 1:**
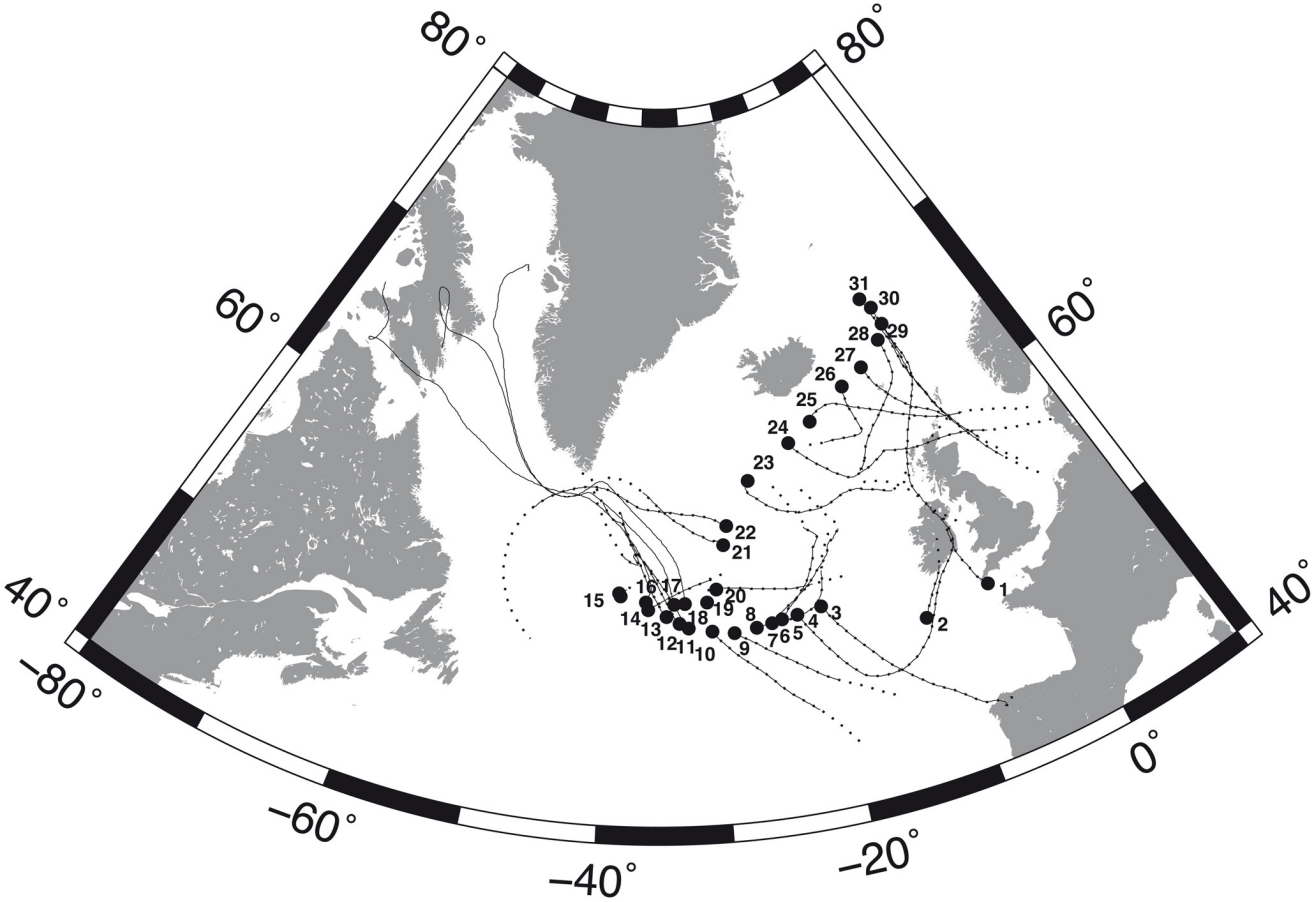
**Sampling sites (black circles) along the MEDEA-II cruise and forward trajectories of the air masses during the calculated time for remaining prokaryotic (continuous lines) and eukaryotic (broken lines) loads of 10%**.

Samples of airborne microbes were collected at different locations in the North Atlantic Ocean during the MEDEA-II cruise on board R/V *Pelagia*, from mid-June to mid-July 2012. Thirty-one aerosol samples were collected along the cruise track covering a range of approximately 17 latitudinal degrees from 49.8° to 67°N (Figure 1).

Air sampling was carried out using a commercially available cyclonic collector (Coriolis-Δ, Bertin Technologies; Carvalho et al., 2008) placed at bow-side at ~10 m height over sea level at the top deck of R/V *Pelagia*. The inlet piece and all the pieces in contact with the sample and the collection liquid were cleaned before every sampling by immersion in diluted HCl (0.5N) for 12 h, rinsed with freshly produced 0.2 μm filter-sterilized Milli-Q water to remove microbial contamination and carried to the sampler covered in clean Ziploc bags. Samples were collected only when the ship was steaming forward and into the wind according to underway measurements of relative wind direction and speed to avoid shipborne contamination. The collection liquid was prepared fresh every day by sterile filtration (0.2 μm) and consisted of Milli-Q water containing 0.005% Triton X-100. Air was drawn into the sampler at 300 l per minute for 6 h (equivalent to 108 m^3^), and the collection liquid was refilled continuously by the system at a rate sufficient to match the loss of liquid by evaporation and re-aerosolization measured during a previous 10 min run. Daily, a field blank was collected by sampling air for 2 min (equivalent to 0.6 m^3^).

Samples were processed immediately after collection and the volume adjusted to 15 ml with additional collection liquid if necessary. Five milliliter aliquots of sample were fixed with formaldehyde (2% final concentration) for 10 min, stained with DAPI (4′, 6- diamidino-2-phenylindole, 1 μg ml^−1^ final concentration) and subsequently filtered onto black 0.2 μm pore size polycarbonate filters (Millipore), mounted on microscope slides and stored frozen for later analysis of the abundance of prokaryotes and unicellular eukaryotes. DAPI-stained samples were examined using a *Leika* DM 1000 epifluorescence microscope equipped with an HBO 50 mercury arc lamp and a filter cube containing a 360/40 BP excitation filter, a 400 nm dichromatic mirror and a 470/40 BP emission filter. DAPI bound to DNA results in bright blue fluorescence at ~390 nm when excited with 365 nm light while DAPI bound to other materials appears as weak yellow or non-fluorescent (Porter and Feig, 1980). Thus, biological (DNA-containing) particles can be readily discriminated from inorganic particles and organic debris using this setup. DNA containing particles approximately smaller than 1 μm and uniformly stained (no clearly defined nucleus) were counted as prokaryotes while larger cells presenting clearly defined, brightly stained nuclei were counted as unicellular eukaryotes (Sherr et al., 1993) to a point.

No traces of multicellular eukaryotic tissue were found in any of the samples. Because of the highly variable abundance of airborne organisms in our samples, we established a counting strategy aimed at providing high precision counts at very low abundances assuming a Poisson distribution. At least 150 particles of interest (bacteria or unicellular eukaryotes) were counted per sample in at least 12 different fields. In samples with very low abundances like field blanks where 150 particles could not be detected, at least 150 fields were counted. This strategy allowed us to ensure a good overall precision (<10% relative standard deviation) even in those samples with very low abundances. Additionally, we determined bacterial abundance in surface seawater by flow cytometry of SYBRGreen I stained samples (Marie et al., 1997). Seawater was collected from the mixed layer with Niskin bottles mounted on a rosette sampling system.

### Accounting for loss of particles by re-aerosolization

One of the caveats of liquid-based samplers is that some of the sample will be inevitably re-aerosolized and carried away by the air flowing through the system (Willeke et al., 1998; Riemenschneider et al., 2010). Thus, we quantified the loss of 1 μm-size particles over time in our collector using the conditions of air flow as in the field sampling. We did so by adding 1 μm diameter fluorescently labeled polystyrene microspheres (Invitrogen) to the collection cups at the onset of the run (~250,000 beads ml^−1^) and evaluating the abundance of the beads remaining at 1 h intervals using flow cytometry. Since the volume in the collection cup is kept constant, we can assume that the collection liquid is aerosolized at a constant rate and becoming more diluted over time. Thus, we can model the number of particles N (particles ml^−1^) in the liquid over time as

(1) $$\frac{dN}{dt}\;=\;k\cdot N$$

which for an initial abundance *N*_0_ = *N*(*t* = 0) can be solved as the exponential function

(2) $$N(t)\;=\;N_{0}\cdot e^{k\cdot t}$$

Fitting the measured abundances of fluorescent particles in the sampler over time by nonlinear regression to Equation (2), we obtained an excellent fit (*R*^2^ = 0.993) and an estimate of *k* = (−0.770 ± 0.049) h^−1^ (mean values ±standard error).

Thus, assuming that microbial abundance in the air is constant over the sampling period, microbial particles (prokaryotic or eukaryotic) enter the sampler at a constant rate c. Then the accumulation of particles in the sampling cup can be expressed as

(3) $$\frac{dN}{dt}\;=\;-0.7701N+c$$

which for an initial number of particles in the sampling cup *N*(field blank) gives

(4) $$N(t)\;=\;1.29853c+0.999998N_{0}\cdot e^{-0.7701t}\;-1.29853c\cdot e^{-0.7701t}$$

where *N*(*t*) is the observed microbial abundance (particles ml^−1^) in the collected sample and *N*_0_ the observed abundance in the field blank. From this equation we obtained the rate of entry of microbial particles in the sampling cup (c, particles ml^−1^ h^−1^) as

(5) $$c=\frac{0.999998N_{0}\cdot e^{-0.7701t}\;-N(t)}{1.29853e^{-0.7701t}\;-1.29853}$$

Our standard sampling conditions (300 l of air per minute for 6 h collected in a final volume of 15 ml) correspond to 1.2 m^3^ of air sampled per ml of sampling liquid per hour (1.2 m^3^ ml^−1^ h^−1^). Thus, the abundance of microbes in the air *C*_*air*_ is given by,

(6) $$C_{air}\;=\;\frac{c}{1.2}$$

Using the value of c from Equation (5), we calculated microbial abundance in the air using the abundances observed in the sample and the field blank and the sampling time *t* using the following expression

(7) $$C_{air}\;=\;\frac{0.641751N_{0}\;-0.641751e^{0.7701t}\;\cdot N(t)}{1-e^{0.7701t}}$$

where *C*_*air*_ is the abundance of microbes in the air (particles m^−3^), *N*(*t*) and *N*_0_ are the microbial abundances in the sample and in the field blank, respectively, (microbes ml^−1^) and *t* is the collection time (hours).

### Microbial load in the boundary layer

The microbial loads (Q; particles per m^2^ of ocean surface), representing the concentration of microorganisms integrated over the atmospheric Boundary Layer Height (BLH), were calculated as the product between the microbial abundance in the air (*C*_*air*_), and the BLH as given by

(8) $$Q\;=\;C_{air}\cdot BLH$$

assuming that the abundance of microorganisms is rapidly homogenized throughout the BLH by turbulent mixing (Lewis et al., 2004). Although there is a gradient in particle concentrations next to the surface of the ocean, gravitationally induced vertical gradients are negligible for small particles (Hoppel et al., 2002) and field measurements show that at moderate wind speeds such as those observed in our study, small particles become already well mixed at heights lower than 10 m (Piazzola and Despiau, 1997). The BLH values, computed by Troen and Mahrt (1986), were extracted from the Analysis from the European Center for Medium-range Weather Forecasts (ERA-Interim; Dee et al., 2011) and averaged from the fields every 3 h during 2 days previous to the observations and over an horizontal area that contained the air mass.

### Conceptual framework

**Figure 2:**
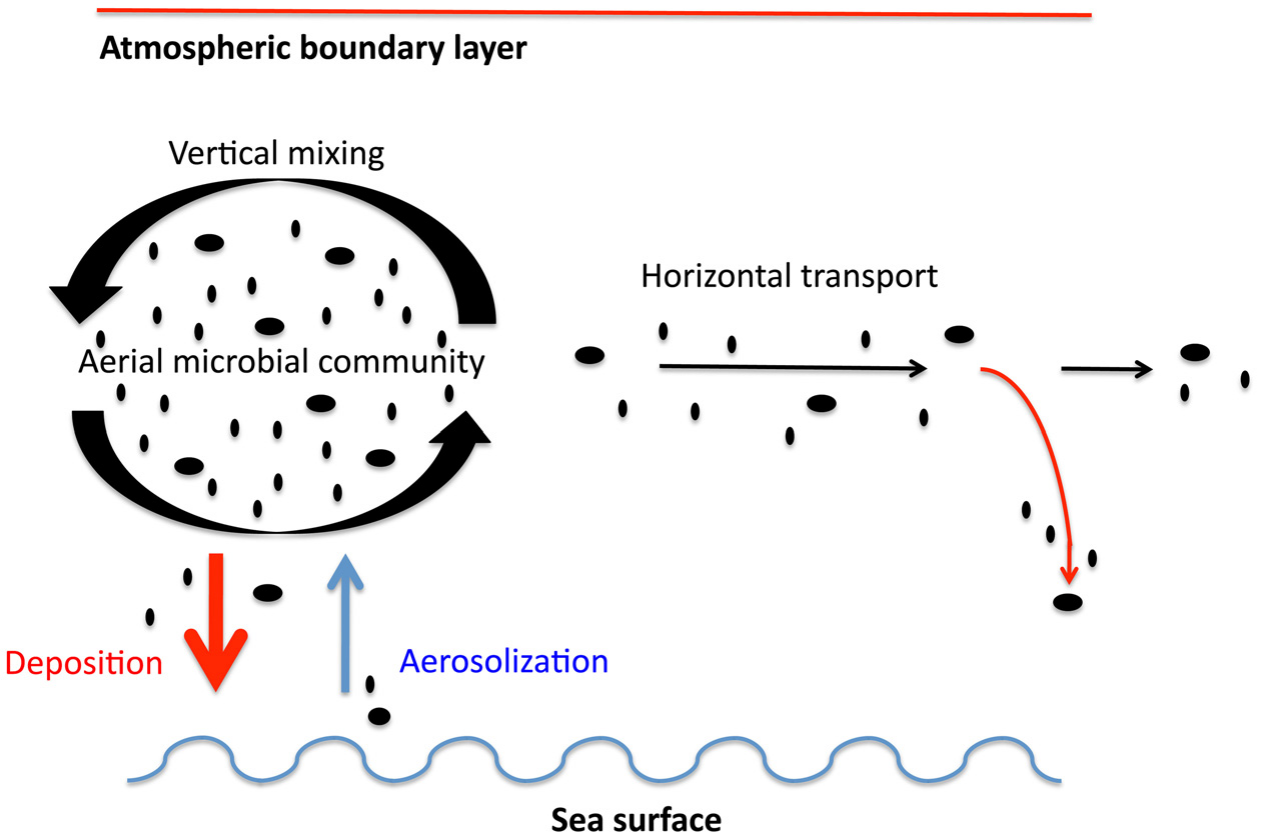
**Processes interacting between atmosphere and ocean taken into account in this work**. Vertical mixing is assumed as a process that generates a homogeneous boundary layer. Aerosolization, deposition and transport are estimated values assuming a homogeneous boundary layer.

We considered two major processes, namely aerosolization from the ocean to the atmosphere and deposition from the atmosphere into the ocean as depicted in Figure 2. Direct flux measurements such as those performed on land (Lindemann et al., 1982; Lighthart and Shaffer, 1994) require sampling at fixed heights and are extremely challenging to implement at sea due to the disturbance caused by the constant rolling of the ship. Also, direct flux measurements provide only the net flux, but separate characterization of both aerosolization and deposition is more relevant for dispersal processes since the microbes entering and leaving the ocean at a given location may not belong to the same taxonomic groups. Therefore, we have calculated deposition and spray fluxes using variables that can be accurately measured on a ship and common parameterizations from the literature. Aerosolization promoted by wind shear over the sea surface was estimated from microbial abundance in surface waters and wind speed. Dry deposition of airborne microbes was estimated from meteorological conditions (wind speed, temperature and relative humidity) and microbial properties such as size and density. Transport over the ocean was estimated using a particle trajectory model (Draxler and Rolph, 2013) and residence times derived from microbial loads and deposition rates. Other processes, such as wet deposition, mass exchange at the top of the boundary layer or particle diffusion, were not taken into account in this study.

### Dry deposition of airborne microorganisms onto the ocean

**Figure 3:**
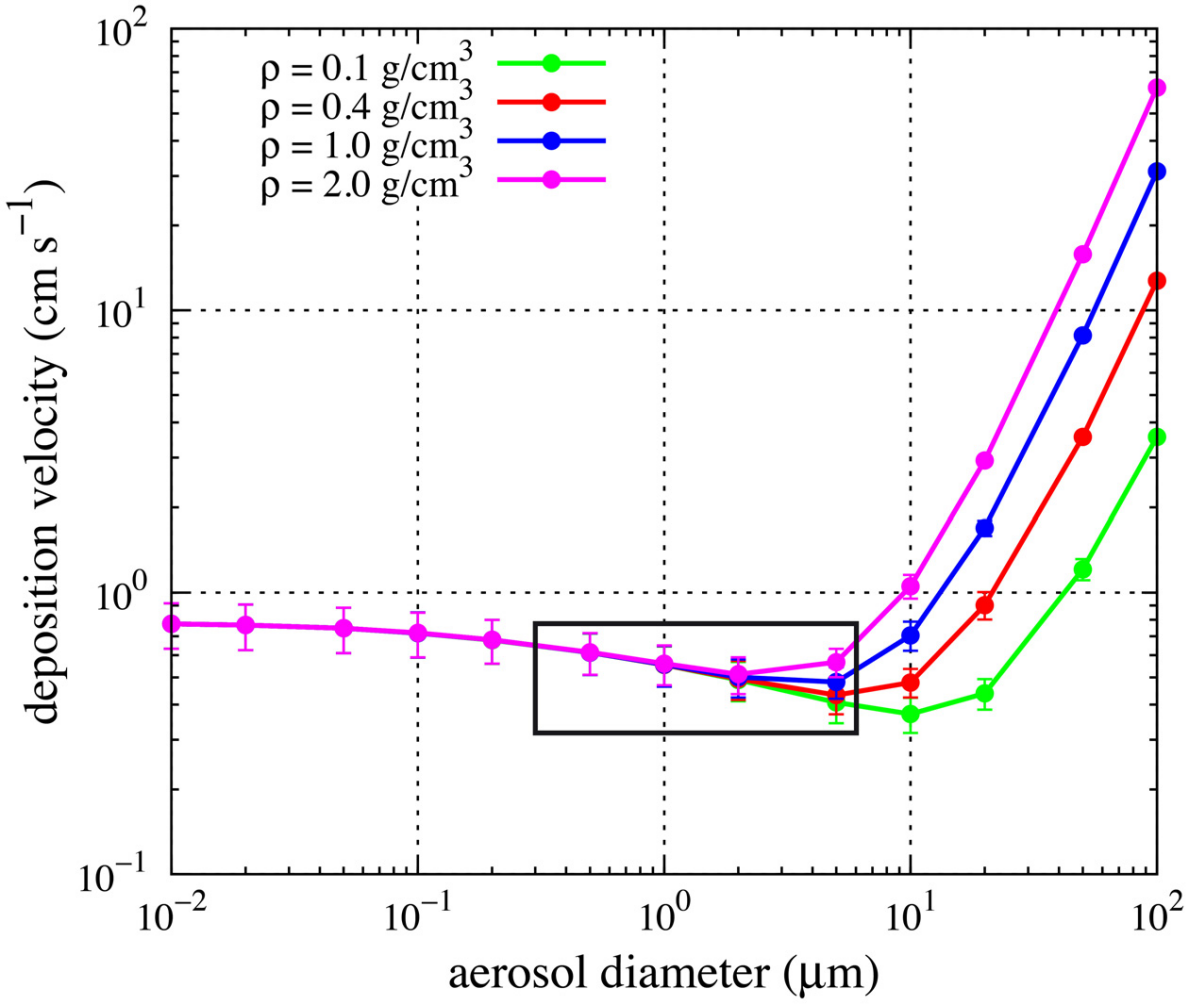
**Dependence of deposition velocities on particle density and diameter**. Points and error bars indicate mean and standard deviations of the estimate using the observed values of humidity, temperature and wind speed at the different sampling stations in this study. The box indicates the range of diameters relevant for this study.

In this study we focused only on dry (not associated to snow or rainfall) deposition. Thus, the deposition velocity (*v*_*d*_, m s^−1^) of airborne material can be parameterized as a function of the diameter and density of the particle and the atmospheric conditions (wind speed, temperature and humidity) (Williams, 1982). The deposition flux (*Fd*) can be estimated from *v*_*d*_ as

(9) $$Fd\;=\;v_{d}\;\cdot C_{air}$$

where C_air_ is the air concentration of the particles, as described by Jurado et al. (2004 and Supplementary Material therein). The values of the atmospheric parameters necessary to compute *v*_*d*_ (wind velocity, temperature, atmospheric pressure and humidity) were extracted as 2-min average values from the continuous recording of the meteorological station installed on board RV Pelagia. The mean value of the meteorological observations recorded during the 6 h of sampling was used to compute *v*_*d*_. We assumed a density of 1.1 g cm^−3^ for biological particles (Bakken and Olsen, 1983) and a mean diameter of 0.5 and 5 μm for prokaryotes and eukaryotes, respectively, as determined by epifluorescence microscopy. Anyway, deposition velocities were largely dependent on wind speed while assumptions regarding density and particle diameter had little effect on the calculated deposition velocity as shown in Figure 3 for the range of particle diameters considered and densities between 0.1 and 2 g cm^−1^.

### Aerosolization of marine microbes into the atmosphere

Surface winds induce the formation of whitecaps and the subsequent generation of sea spray droplets, which carry sea salts and microbes into the atmosphere. Following Andreas (1998), the total volume flux of seawater spray into the atmosphere ($\overset{•}{V_{T}}$, m^3^ m^−2^ s^−1^) is computed as:

(10) $$\overset{•}{V_{T}}\;=\;\frac{4\pi }{3}\int_{4}^{15}r_{0}^{3}\;\frac{dF}{dr_{0}}dr_{0}$$

where *dF/dr*_0_ is the spray generation function for droplets of an initial radius *r*_0_ and it is computed as a function of the wind speed or the wind friction velocity (u^*^). Different formulations of *dF/dr*_0_ have been proposed (cf. O'Dowd and de Leeuw, 2007) depending on the droplet radius and wind speed. For our calculations, we chose the formulation of Blanchard (1963) and Gathman (1982) as corrected by Andreas et al. (1995) using *in situ* observations of wind speed and humidity. This formulation is valid for wind speeds between 5 and 15 m s^−1^, well suited for the wind speeds observed during the MEDEA-II cruise (average ~8 m s^−1^). We also constrained the estimation considering only droplets in the range of radii from 0.2 to 10 μm as most microbial cells will be larger than 0.1 μm and droplets >10 μm are not likely to remain airborne when the wind speed is lower than 9 m s^−1^ (Andreas et al., 1995). The choice of parameterization has little effect on the calculated spray fluxes, since other parameterizations of sea spray generation yield similar values for the particle size range between 0.1 and 10 μm for wind speeds of 8 m s^−1^ (De Leeuw et al., 2011).

The flux of microbes associated to sea spray (*Fs*) was thus computed by multiplying the total volume flux $\overset{•}{V_{T}}$ by the microbial abundances in sea surface water samples. Thus, the sea spray flux is comparable to the deposition flux (in terms of units) computed in the previous section. There are indications that microbial abundances in spray droplets may be higher than in surface waters (Blanchard et al., 1981), thus our estimate of spray flux calculation may be a minimum one. We did not attempt to correct for this enrichment since the reported enrichment factors vary enormously from day to day (Aller et al., 2005).

### Remaining microbial load in the atmosphere and potential for dispersal

The microorganisms suspended in the atmosphere are deposited according the Equation (9) and as determined by the following expression

(11) $$Fd\;=\;-\frac{dQ}{dt}$$

where *Q* corresponds to the microbial load from Equation (8). Equating both Equations (9, 11), where *C*_*air*_ is expressed as determined from Equation (8), and then integrating we obtained the remaining microbial load of suspended microorganisms at any given time as

(12) $$Q(t)=Q_{0}\cdot e^{\frac{-v_{d}\;\cdot \;t}{BLH}}$$

where *Q*(*t*) is the remaining microbial load, *Q*_0_ initial microbial load and *t* corresponds to time.

Forward trajectories were simulated using the HYSPLIT model (Draxler and Rolph, 2013) for particles originally situated at a height of 10 m above sea level at the sampling locations for the time necessary to reduce the microbial load to 10% of the original value as estimated from Equation (12). The distance traveled by the airborne microbes corresponds to the length of the estimated forward trajectories.

## Results

### Abundance of airborne prokaryotes and eukaryotes over the north atlantic ocean

The airborne prokaryotic abundance ranged from 2782 to 19,132 prokaryotes m^−3^ (average 8020 cells m^−3^) while the abundance of airborne eukaryotes ranged from 202 to 12,805 eukaryotes m^−3^ (average 1998 cells m^−3^, Table 1).

**Table 1:** **Abundance, derived spray and deposition fluxes and estimated load of airborne microorganisms over the North Atlantic Ocean obtained in this study (range is given in the upper row and averages in the lower row)**.

	**Abundance (cells m^−3^)**	**Estimated spray flux (cells m^−2^ s^−1^)**	**Estimated deposition flux (cells m^−2^ s^−1^)**	**Estimated load (cells m^−2^)**
Prokaryotes	2782–19,132 (8020)	9.25–100.64 (42.5)	15.30–141.43 (49.0)	393,777–16.082,289 (3.651,764)
Eukaryotes	202–12,805 (1998)	0.01–0.1 (0.04)	1.05–64.02 (9.85)	60,571–3.598,525 (759,887)

**Figure 4:**
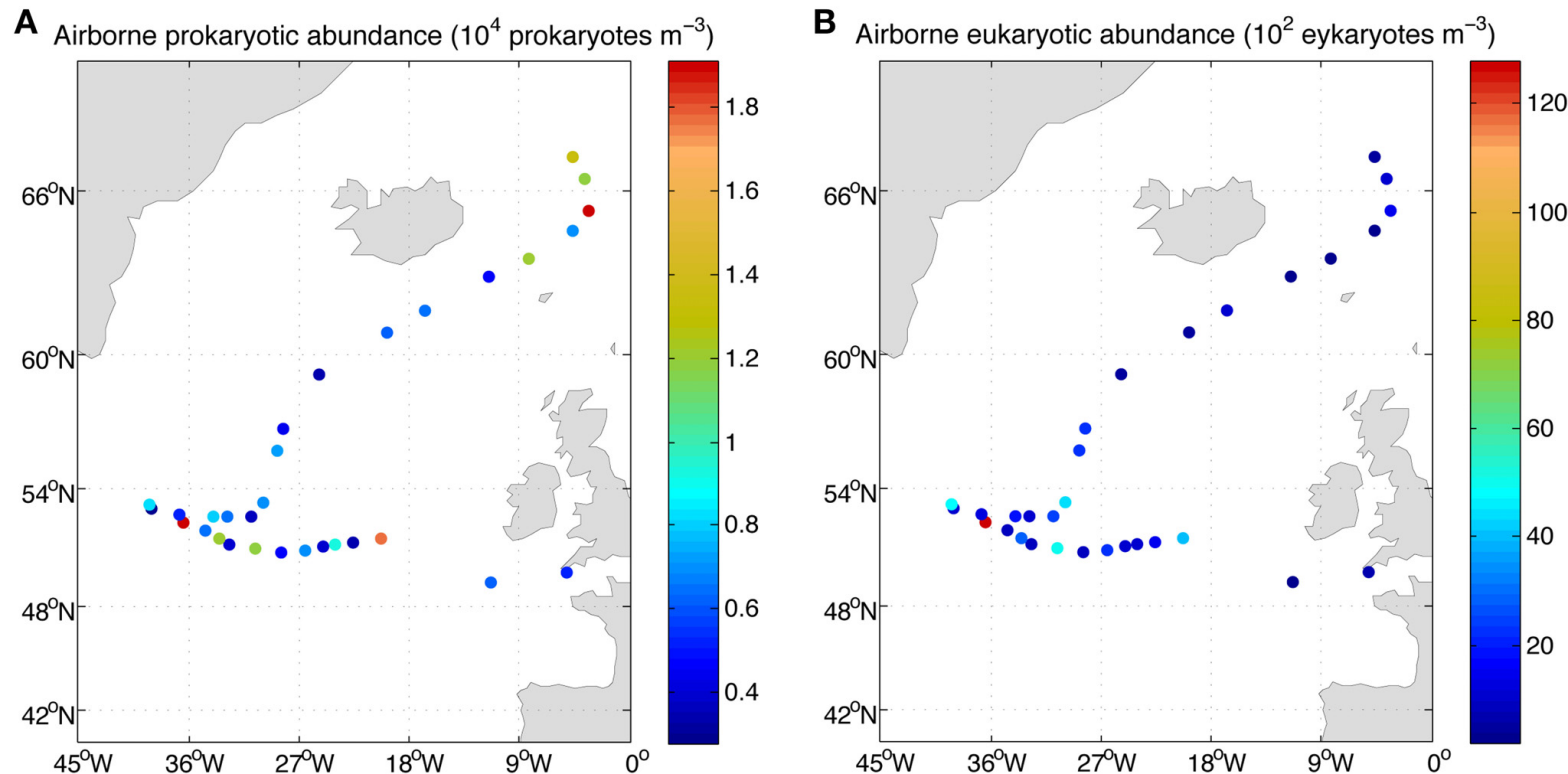
**Concentration of airborne prokaryotes (A) and eukaryotes (B) over the eastern North Atlantic along the MEDEA-II cruise**.

Lowest abundances of airborne bacteria were detected around 60°N and eukaryotic abundances generally decreased toward the North (Figure 4). Integrating the abundances of prokaryotes and eukaryotes over the height of the boundary layer resulted in an estimated microbial load ranging between 0.4 × 10^6^ and 16 × 10^6^ prokaryote cells m^−2^ and 6 × 10^4^ to almost 3.6 × 10^6^ eukaryote cells m^−2^ (Table 1). The height of the boundary layer showed large variations along the sampled track ranging between 93 and 919 m, with the lowest heights related to strong subsidence in the upper atmosphere and presence of fog, but the general pattern of the estimated microbial load was mainly determined by the microbial abundance.

### Aerosolization and deposition fluxes and transport of airborne microbes

**Figure 5:**
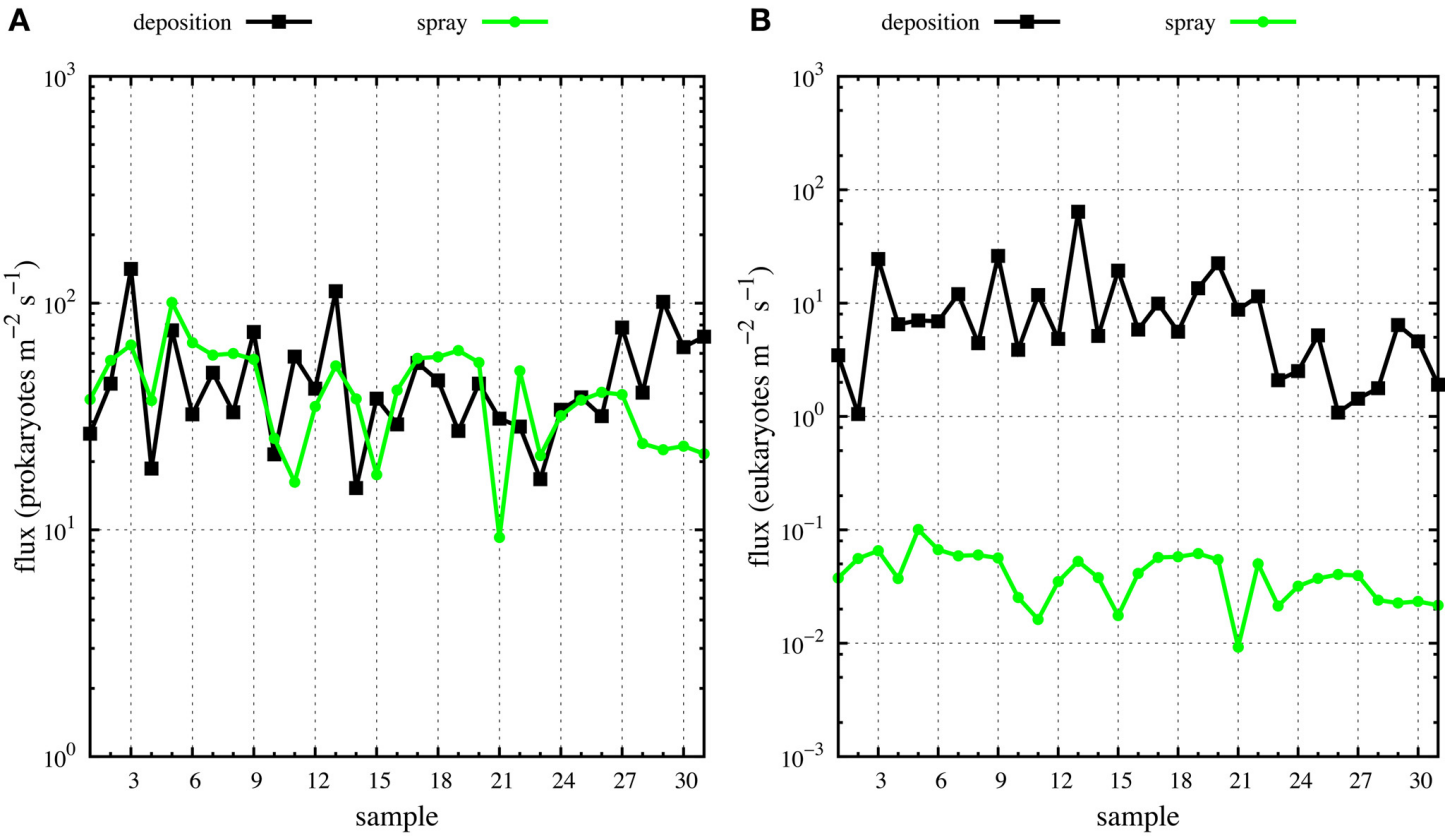
**Deposition (black dots) and spray (green dots) fluxes of airborne prokaryotes (A) and eukaryotes (B) along MEDEA-II cruise**.

Mean wind speed during the MEDEA-II cruise was 7.9 ± 3.3 m s^−1^ and the prokaryotic abundances in surface seawater samples ranged from 8 × 10^5^ to 2.3 × 10^6^, resulting in averaged emissions with spray of 42.5 prokaryotes m^−2^ s^−1^ (range 9.25–100.64 prokaryotes m^−2^ s^−1^). The abundance of small eukaryotic cells in surface seawater was not measured but we calculated order-of-magnitude estimates ranging from 0.01 to 0.1 eukaryotes m^−2^ s^−1^ (average 0.04 eukaryotes m^−2^ s^−1^, Figure 5) assuming a constant relationship between the abundance of heterotrophic bacteria and small protists in surface seawater (Zubkov et al., 2007).

**Figure 6:**
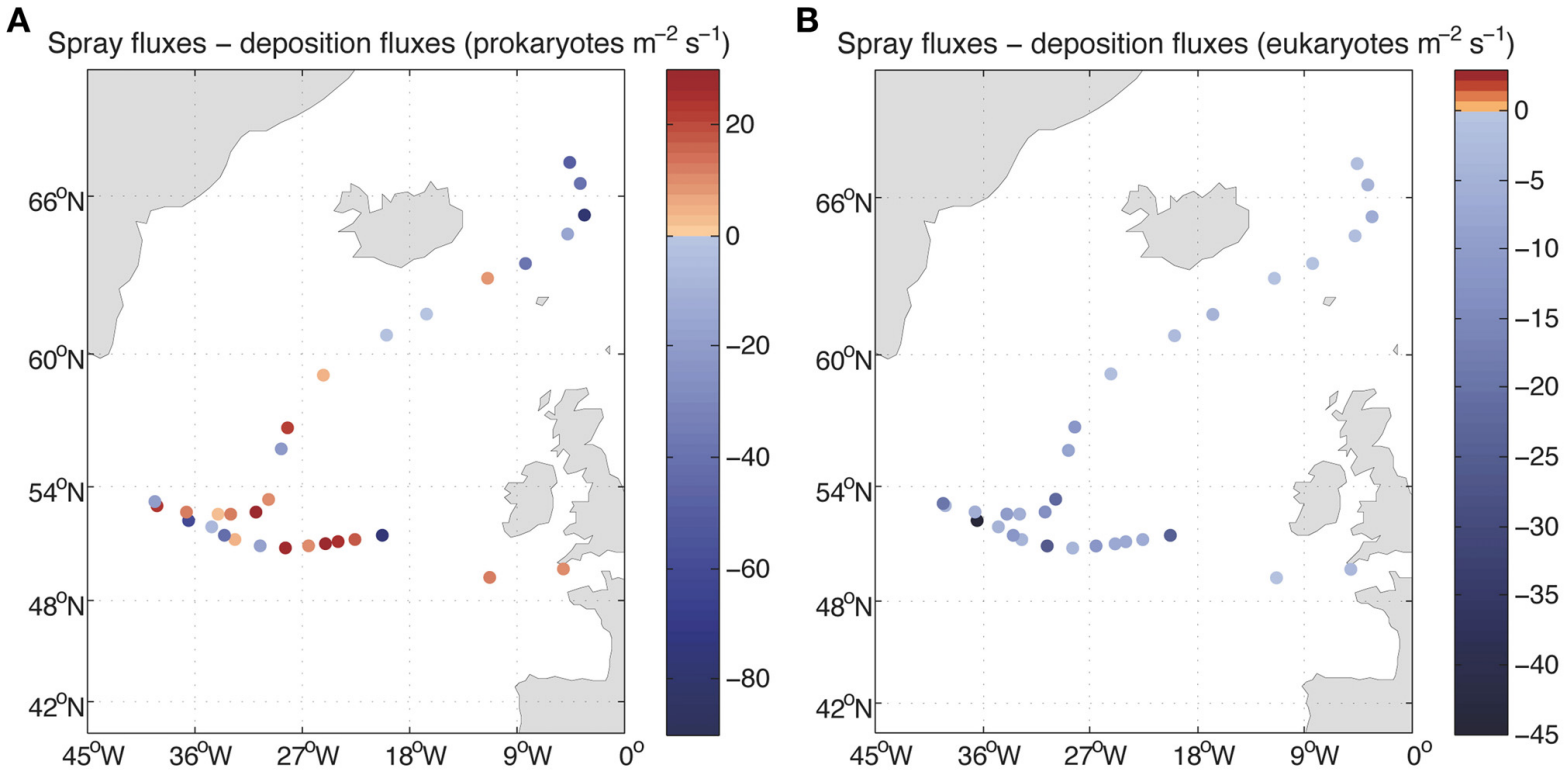
**Net fluxes of prokaryotes (A) and eukaryotes (B) between air and sea**. The values lower than zero correspond to net fluxes from the atmosphere into the ocean.

The derived average deposition fluxes were 49.0 prokaryotes m^−2^ s^−1^ and 9.85 eukaryotes m^−2^ s^−1^ ranging from 15.30 to 141.43 prokaryotes m^−2^ s^−1^ and from 1.05 to 64.02 eukaryotes m^−2^ s^−1^ (Figure 5). Net fluxes calculated as spray fluxes minus deposition fluxes (negative values denote net flux into the ocean) averaged –6.49 prokaryotes m^−2^ s^−1^ and –9.81 eukaryotes m^−2^ s^−1^. Net fluxes of prokaryotes were negative in 45% of sampled locations, while the ocean was a net sink for eukaryotes at all stations sampled (Figure 6). The locations where the ocean acted as a net source of microorganisms to the atmosphere were mostly situated south of 60**°**N.

The estimated deposition rates indicated that the time necessary to deposit 50% of the suspended cells was on average 0.6 and 0.7 days for prokaryotes and eukaryotes, respectively, (maximum 1.2 and 1.4 days). Moreover, our calculations also indicated that 10% of the suspended microbes remained airborne after 4 (prokaryotes) and 4.8 days (eukaryotes) at some locations (average 2 and 2.4 days for prokaryotes and eukaryotes, respectively). Moreover, it was estimated that 1% of the sampled microbes were still airborne after up 8 (prokaryotes) and 9.6 days (eukaryotes) on average 4 and 4.8 days, respectively. Modeling forward trajectories for the time required to reduce the microbial load to 10% of the original revealed that 90% of the prokaryotes were deposited over distances between 6308 and 10,337 km, with an average of 8273 km, while 10% of the eukaryotes were still airborne after traveling from 6499 to 11,379 km, with an average of 8623 km (Figure 1).

## Discussion

It is difficult to compare our results to other oceanic estimates since only very few studies have been conducted at open oceanic locations. Indeed, Burrows et al. (2009b) did not report any abundance estimates for remote oceanic locations, simply because these were absent from the literature at that time. Other, more recent studies on airborne bacteria over the ocean derive from locations either influenced by terrestrial sources (Cho and Hwang, 2011) or from high altitudes well above the boundary layer (Smith et al., 2012; Yamaguchi et al., 2012; DeLeon-Rodriguez et al., 2013). The abundance of airborne prokaryotes and eukaryotes reported in our study are between 10 and 100-fold lower than those reported in other studies (Cho and Hwang, 2011; DeLeon-Rodriguez et al., 2013). These differences are probably due to the fact that none of those studies is representative for the atmospheric boundary layer of the open ocean as well as to methodological differences. Even studies developed in the atmospheric boundary layer over open sea are difficult to compare with our data, because the differences among data sets are probably due to the use of different counting methods. For example, airborne microbial abundances reported by Marks et al. (2001) from the Baltic Sea, resulted in one and two order of magnitude lower than those reported in our study for eukaryotes and prokaryotes, respectively, probably due to the low efficiency of cultivation-dependent counts. Conversely, Cho and Hwang (2011) reported abundances of airborne bacteria ranging from 0.7 to 1.2 × 10^5^ cells m^−3^ using epifluorescence microscopy, one order of magnitude higher than our values, probably due to the fact that their samples come from a nearly-enclosed sea heavily influenced by land. Similarly, Matthias-Maser et al. (1999) reported average values of 5.9 × 10^5^ biological particles in an area affected by African dust episodes. Also, the higher abundances obtained by microscopic counts reported by DeLeon-Rodriguez et al. (2013) correspond to high altitude samples and influenced by hurricanes. However, these authors reported bacterial abundances exceeding their own estimates of total particles in the sample. The authors explained these discrepancies by the low accuracy of their bacterial counts resulting from the very low abundance in their samples. Indeed, these authors counted only 16 microscopic fields while we estimated that up to 150 fields must be counted to obtain reliable results below the 10% relative standard deviation threshold in low abundance samples (see Materials and Methods). Yet, qPCR estimates of bacterial abundance reported in the same studies (Cho and Hwang, 2011; DeLeon-Rodriguez et al., 2013) result in much lower abundances similar to the range of 10^3^–10^4^ cells m^−3^ that we found over the North Atlantic Ocean although these estimates are expected to yield overestimates of microbial abundance due to the presence of multiple copies of the rRNA operon. Hence, the abundances of airborne microbes reported here for the open North Atlantic Ocean are the first estimates reported thus far for open-oceanic locations.

Collecting airborne microbes in liquid systems might result in large sample losses by re-aerosolization such as those reported for impingers (Riemenschneider et al., 2010), swirling liquid collectors (Willeke et al., 1998) or those reported in this paper for our cyclonic sampler. However, these losses can be corrected quite accurately (see Materials and Methods). Moreover, liquid samples have the advantage that they can be processed using standard methods developed for aquatic microbiology. Contamination may, however, be an issue in liquid samples, but microbial abundances in field blanks were always much lower than those in the samples indicating that the microbes were actually derived from the atmosphere. Moreover, due to the exponential dilution observed over time in the experiments with fluorescent microspheres, the expected contribution of any contaminating microbes present at the onset of the collection on the final estimated abundance is negligible. Collection efficiencies vary widely among different samplers for different particle sizes (Nevalainen et al., 1992; Dybwad et al., 2014). It has been reported that for our Coriolis sampler, the physical sampling efficiency for particles in the size range of bacteria (0.2–1.0 μm in diameter) is around 50% but close to 100% for particles larger than 4 μm (Carvalho et al., 2008; Dybwad et al., 2014). We did not attempt to correct for this since the reported low collection efficiencies are mainly due to re-aerosolization for which we already corrected and also because many of the airborne bacteria can be attached to larger particles.

The forward trajectories of the air masses sampled here over the North Atlantic Ocean are consistent with global patterns of atmospheric circulation, with a high-pressure system over Greenland generating northerly winds in the north section of MEDEA-II cruise. A low-pressure system over the Atlantic Ocean (below 60**°**N) generated southerly and westerly winds in the southern region of MEDEA-II cruise. These modeled trajectories, together with our estimates of residence time, suggest that most airborne bacteria at the stations sampled were of oceanic origin.

Estimations of airborne microbial abundance over the open ocean are needed to resolve the biology of the atmosphere, the Earth's biome where life is most diluted but that, as demonstrated for other similarly diluted constituents (e.g., nitrogen, pollutants or gasses), plays a fundamental role in transport and connectivity across biomes (Uematsu et al., 1983; Krishnamurthy et al., 2010). Determining the concentration and loads of atmospheric microbes is an important step, and the estimates provided here suggest that microbes are diluted in the atmosphere more than 9 or 11 orders of magnitude relative to their concentration in seawater or soils (Whitman et al., 1998). This could lead to the conclusion that airborne bacteria are unimportant and can be neglected. Yet, the abundance of airborne microbes may be a misleading indicator of the importance of this compartment, as the atmosphere may play a major role in the dispersal of microbes, in the connectivity and the maintenance of diversity in the surface ocean or in regulating climatic processes through the role of airborne bacteria as nuclei of accretion for cloud and ice formation. Hence, while diluted relative to the marine or soil compartment, the estimated microbial load over the height of the boundary layer averaging 3.6 × 10^6^ prokaryotes m^−2^ and 7.6 × 10^5^ eukaryotes m^−2^ represents a formidable seed bank hovering over the North Atlantic Ocean. The net fluxes are relatively small (Figure 6) compared to those reported for land locations. Bacterial net flux measurements over a chaparral, oscillated over the day between −8 and 5 CFU m^2^ s^−1^ (Lighthart and Shaffer, 1994) while only upward fluxes up to 553 CFU m^2^ s^−1^ were reported for vegetated agricultural soils (Lindemann et al., 1982), if we consider that cultivable bacteria may be about 1% of the total bacterial load, our net bacterial fluxes could be up to 4 orders of magnitude lower. However, the microbes entering and leaving the surface ocean are not necessarily the same and thus, the calculated air-sea exchange of microbes shows that it is a highly dynamic process. Averages of 3.6 × 10^6^ prokaryotes and 3.7 × 10^3^ eukaryotes per square meter leaving the ocean into the atmosphere every day were calculated in this study, while an average of 4.2 × 10^6^ prokaryotes and 8.5 × 10^5^ eukaryotes per square meter enter the surface ocean from the atmosphere every day. These values evidence a rapid turnover of atmospheric microbes but also a high dispersal capacity since 10% of the microorganisms present in a given sample will still be airborne on average after 2 days in the case of prokaryotes and 2.4 days for eukaryotes and 1% will still be airborne on average after 4 days (prokaryotes) and 4.8 days (eukaryotes). In other words, 90% of the microbes present in the original air sample will be deposited in about 2 days over large stretches of the ocean >8000 km long.

In conclusion, we found atmospheric microbial abundances in the boundary layer over the North Atlantic Ocean ranging from 10^3^ to 10^4^ prokaryotes m^−3^ and from 10^2^ to 10^4^ eukaryotes m^−3^, but supporting daily air-sea exchanges in the order of millions of prokaryotes and thousands of unicellular eukaryotes per square meter of oceanic surface. This limited dataset provides a first snapshot of the microbial abundances and fluxes over the North Atlantic Ocean during our cruise. Additional efforts are needed to assess the temporal variability and the magnitude of these processes in this and other regions of the ocean. Calculations based on current parameterizations are crude and should be considered as order-of-magnitude estimates. Nevertheless, our data point to a rapid exchange of microbes between the atmosphere and the surface ocean, which is not apparent from abundance data only. This rapid flux could be of major importance for the dispersal of marine microbes and for the maintenance of local diversity over the global ocean.

### Conflict of interest statement

The authors declare that the research was conducted in the absence of any commercial or financial relationships that could be construed as a potential conflict of interest.
