# Corrigendum: Resolving the abundance and air-sea fluxes of airborne microorganisms in the North Atlantic Ocean

**DOI:** 10.3389/fmicb.2017.01971

**Published:** 2017-10-13

**Authors:** Eva Mayol, María A. Jiménez, Gerhard J. Herndl, Carlos M. Duarte, Jesús M. Arrieta

**Affiliations:** ^1^Department of Global Change Research, Institut Mediterrani d'Estudis Avançats, Consejo Superior de Investigaciones Científicas - Universitat de les Illes Balears, Mallorca, Spain; ^2^Department of Limnology and Bio-Oceanography, Center of Ecology, University of Vienna, Vienna, Austria; ^3^The UWA Oceans Institute, The University of Western Australia, Crawley, WA, Australia

**Keywords:** airborne microbes, microbial dispersal, air-sea exchange, bioaerosols, Atlantic Ocean

We found an implementation error in the calculation of the deposition velocity (*v*_*d*_) which, in turn, affected all the estimated *v*_*d*_-depending parameters (deposition flux, residence time, and traveled distance by microorganisms). Deposition fluxes are now somewhat lower than previously estimated, resulting in residence times and traveled distances longer than those previously estimated. In addition, the spray fluxes were calculated using a spray generation function (*dF/dr*_0_) valid for droplets of radii between 0.5 and 12 μm proposed by Blanchard (1963) and Gathman (1982) as corrected by Andreas et al. (1995). However, in the calculation of *dF/dr*_0_, we exceeded this valid range of radii given that we included droplets with radii from 0.2 μm according to the small size of some microbial cells. Thus, a different formulation of *dF/dr*_0_, developed by Gong (2003), is now used for the estimation of spray fluxes of microbes, which is valid even for small droplets from a radius of 0.07 μm.

Below, we offer a new corrected version of the paragraphs affected by corrections along the text. In addition, we show corrected versions of Figure 1 (forward trajectories according residence times), Figure 3 (deposition velocity values), Figure 5 (spray and deposition fluxes), Figure 6 (Net fluxes), and Table 1. The authors apologize for the errors in the estimates reported in the original manuscript. These corrections only affect the magnitude of some of the reported variables and even though they do not change the scientific conclusions of the article they are reported here for accuracy and reproducibility.

**Figure 1:**
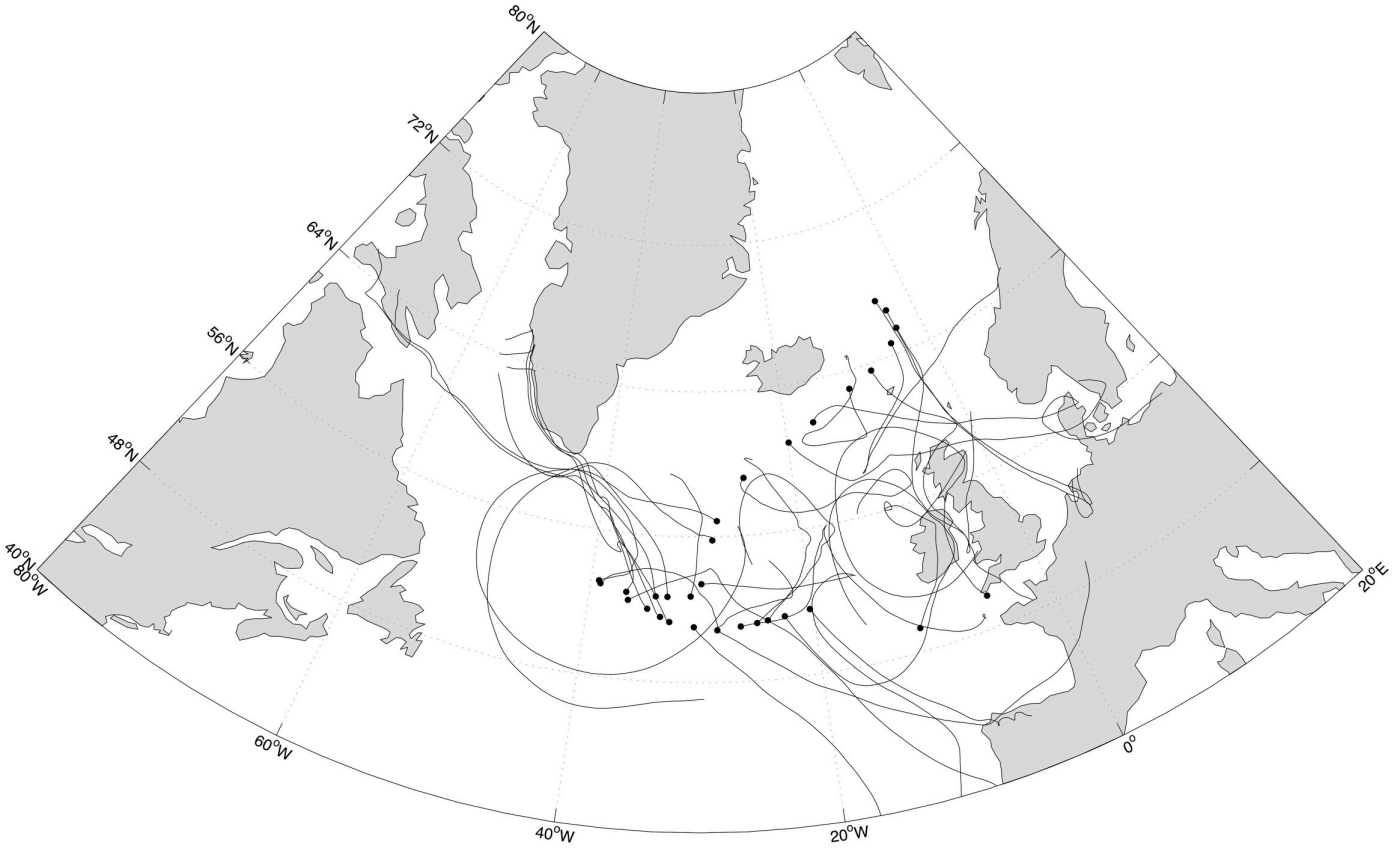
Sampling sites (black circles) along the Medea-II cruise and forward trajectories (black lines) of the air masses during a maximum of 5 days of the calculated time for remaining prokaryotic loads of 50%.

**Figure 3:**
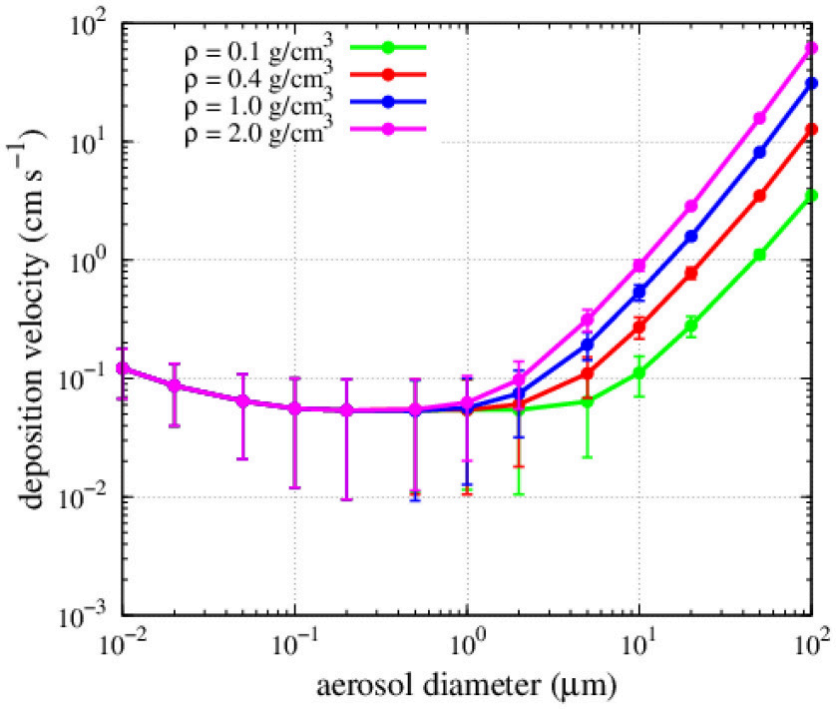
Dependence of deposition velocities on particle density and diameter. Points and error bars indicate mean and standard deviation of the estimate using the observed values of humidity, temperature and wind speed at the different sampling stations in this study.

**Figure 5:**
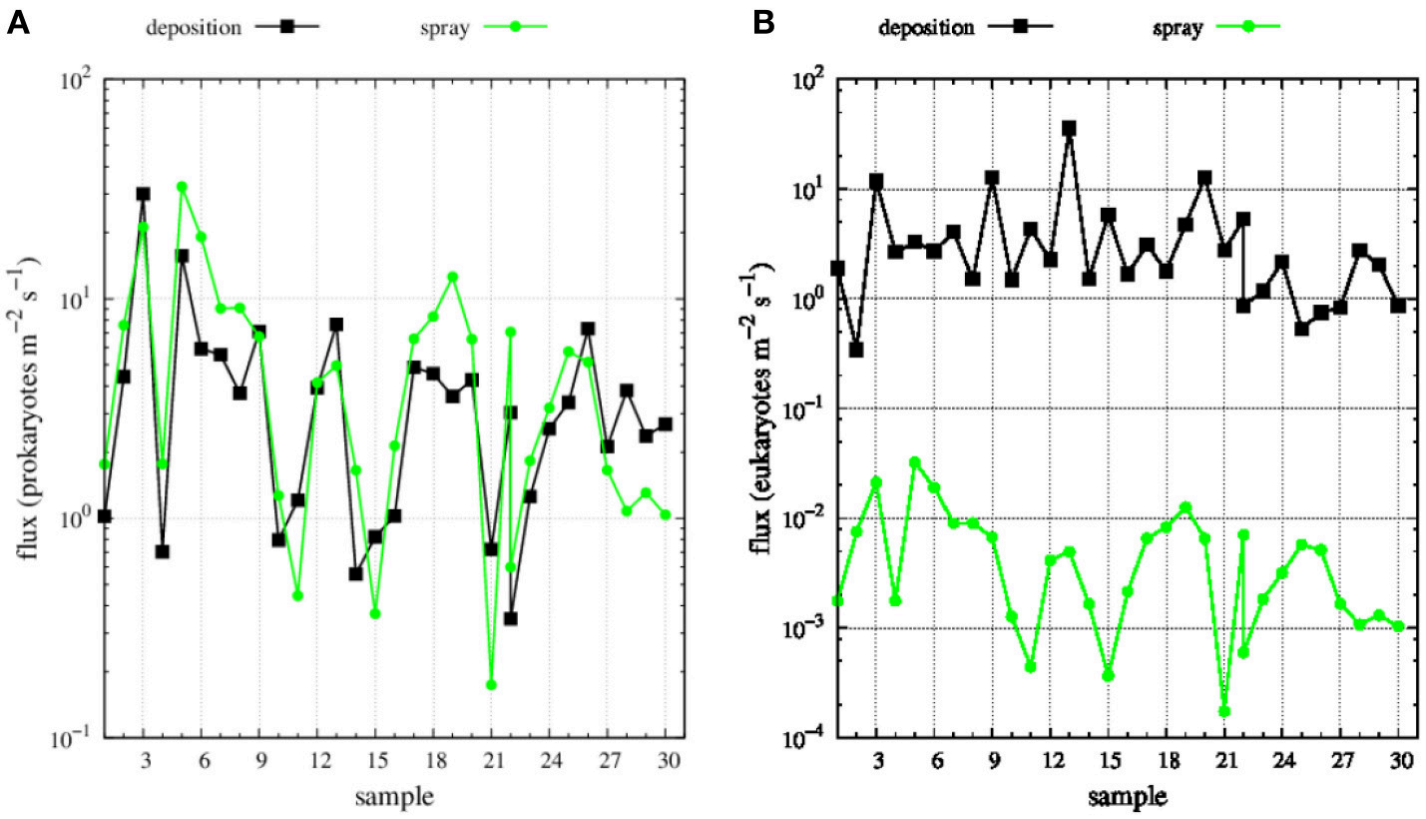
Deposition (black dots) and spray (green dots) fluxes of **(A)** prokaryotes and **(B)** eukaryotes along Medea-II cruise.

**Figure 6:**
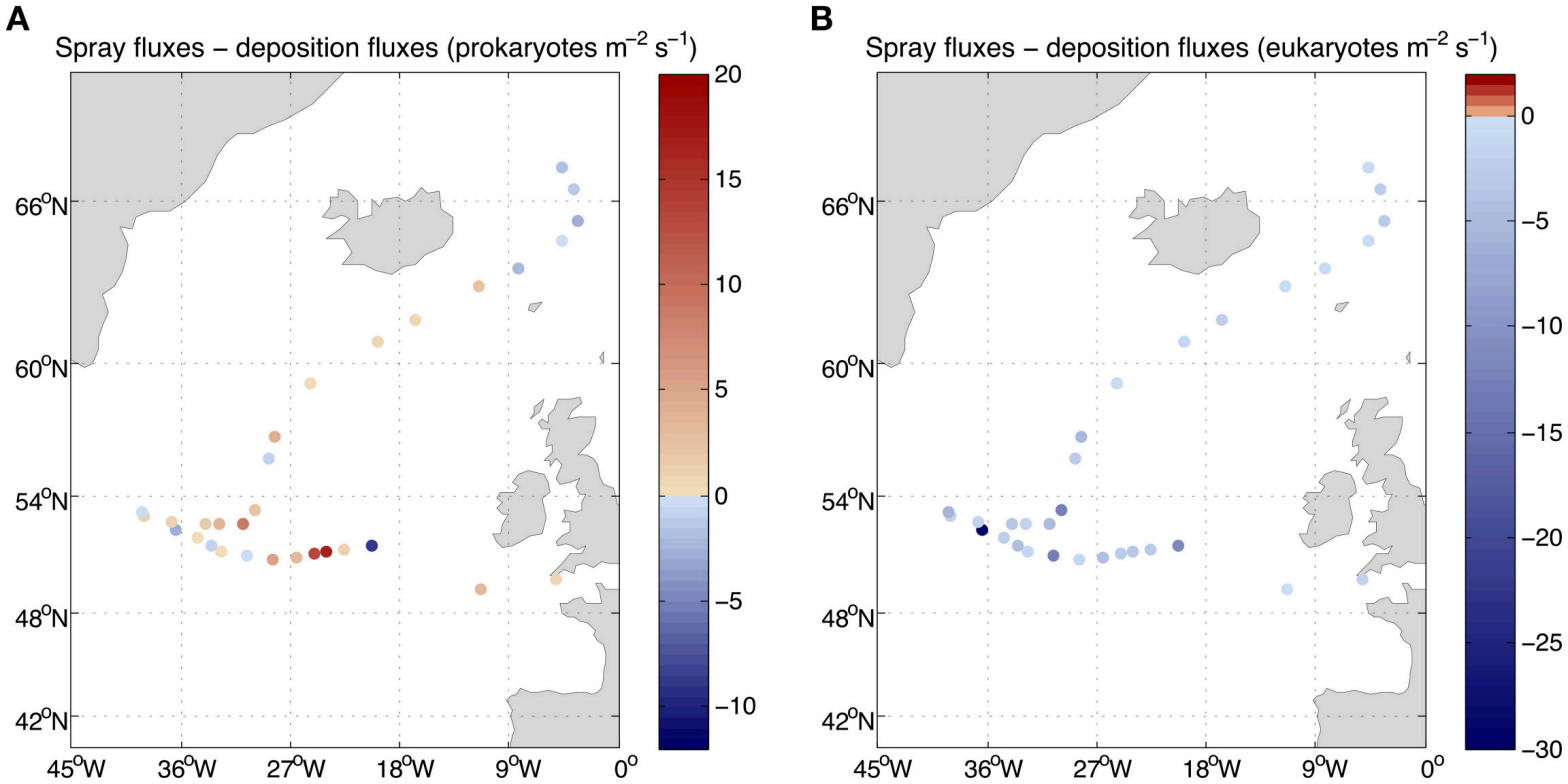
Net fluxes of **(A)** prokaryotes and **(B)** eukaryotes between air and sea. The values lower than zero correspond to net fluxes from the atmosphere into the sea.

**Table 1:** Abundance, estimated spray and deposition fluxes, and estimated load of airborne microorganisms over the North Atlantic Ocean obtained in this study (averages are included in parenthesis).

	**Abundance (cells m^−3^)**	**Estimated spray flux (cells m^−2^ s^−1^)**	**Estimated deposition flux (cells m^−2^ s^−1^)**	**Estimated load (cells m^−2^)**
Prokaryotes	2,782–19,132 (8,020)	0.2–32.5 (6.0)	0.3–30.1 (4.4)	393,777–16,082,286 (3,651,764)
Eukaryotes	202–12,805 (1,998)	0.0002–0.03 (0.006)	0.3–36.0 (4.4)	60,571–3,598,525 (759,887)

In the Abstract, the lower deposition rates calculated result in much longer residence times in the atmosphere; therefore we report the results for 50% of the remaining microbial load instead of 10% in order to keep the back-trajectory analysis within reasonable time frames. The corrected abstract appears below:

Airborne transport of microbes may play a central role in microbial dispersal, the maintenance of diversity in aquatic systems and in meteorological processes such as cloud formation. Yet, there is almost no information about the abundance and fate of microbes over the oceans, which cover >70% of the Earth's surface and are the likely source and final destination of a large fraction of airborne microbes. We measured the abundance of microbes in the lower atmosphere over a transect covering 17° of latitude in the North Atlantic Ocean and derived estimates of air-sea exchange of microorganisms from meteorological data. The estimated load of microorganisms in the atmospheric boundary layer ranged between 6 × 10^4^ and 1.6 × 10^7^ microbes per m^2^ of ocean, indicating a very dynamic air-sea exchange with millions of microbes leaving and entering the ocean per m^2^ every day. Our results show that on average 50% of the microbes detected in the boundary layer were still airborne 12 days later and that they could travel up to 12,000 km before they entered the ocean again. The size of the microbial pool hovering over the North Atlantic indicates that it could play a central role in the maintenance of microbial diversity in the surface ocean and contribute significantly to atmospheric processes.

In the Materials and Methods section, sub-section Aerosolization of Marine Microbes Into the Atmosphere, first paragraph; we have updated this section to reflect that we have now used the formulation of Gong (2003). The corrected paragraph is below:

Surface winds induce the formation of whitecaps and the subsequent generation of sea spray droplets, which carry sea salts and microbes into the atmosphere. Following Andreas (1998), the total volume flux of seawater spray into the atmosphere (VT·VT•, m^3^ m^−2^ s^−1^) is computed as:

$$\begin{array}{l} \text{ VT}\;\cdot =4\pi 3\int^{\text{​}}154\text{r}30\text{dFdr}0\text{dr}0\\ \left(10)\text{VT}•=4\pi 3\int^{\text{​}}415\text{r}03\text{dFdr}0\text{dr}0\;\;\;\;\;(10\right) \end{array}$$

where *dF/dr*_0_ is the spray generation function for droplets of an initial radius *r*_0_ and it is computed as a function of the wind speed or the wind friction velocity (u^*^). Different formulations of *dF/dr*_0_ have been proposed (cf. O'Dowd and de Leeuw, 2007) depending on the droplet radius and wind speed. For our calculations, we chose the formulation of Gong (2003) using *in situ* observations of wind speed and humidity. This formulation is valid for radii between 0.07 and 200 μm, and it is not restricted to specific ranges of wind speeds. We also constrained the estimation considering only droplets in the range of radii from 0.2 to 10 μm as most microbial cells will be larger than 0.1 μm and droplets >10 μm are not likely to remain airborne when the wind speed is lower than 9 m s^−1^ (Andreas et al., 1995). The choice of parameterization has little effect on the calculated spray fluxes, since other parameterizations of sea spray generation yield similar values for the particle size range between 0.1 and 10 μm for wind speeds of 8 m s^−1^ (De Leeuw et al., 2011).

In the Materials and Methods section, sub-section Remaining microbial load in the atmosphere and potential for dispersal, second paragraph; we have updated it to reflect that the microbial load was reduced to 50% of the original value. The corrected section appears below:

Forward trajectories were simulated using the HYSPLIT model (Draxler and Rolph, 2013) for particles originally situated at a height of 10 m above sea level at the sampling locations for the time necessary to reduce the microbial load to 50% of the original value as estimated from Equation (12).

In the Results section, sub-section Aerosolization and Deposition Fluxes and Transport of Airborne Microbes, all three paragraphs have been updated to reflect the corrections to the calculated fluxes, residence times in the atmosphere, and traveled distances of prokaryotes and eukaryotes. The Results section should read:

Mean wind speed during the MEDEA-II cruise was 7.9 ± 3.3 m s^−1^and the prokaryotic abundances in surface seawater samples ranged from 8 × 10^5^ to 2.3 × 10^6^, resulting in averaged emissions with spray of 6.0 prokaryotes m^−2^ s^−1^ (range 0.2 to 32.5 prokaryotes m^−2^ s^−1^). The abundance of small eukaryotic cells in surface seawater was not measured but we calculated order-of-magnitude estimates ranging from 0.0002 to 0.03 eukaryotes m^−2^ s^−1^ (average 0.006 eukaryotes m^−2^ s^−1^, Figure 5) assuming a constant relationship between the abundance of heterotrophic bacteria and small protists in surface seawater (Zubkov et al., 2007).

The derived average deposition flux was 4.4 prokaryotes and eukaryotes m^−2^ s^−1^ ranging from 0.3 to 30.1 prokaryotes m^−2^s^−1^ and from 0.3 to 36.0 eukaryotes m^−2^ s^−1^ (Figure 5). Net fluxes calculated as spray fluxes minus deposition fluxes (negative values denote net flux into the ocean) averaged 1.6 prokaryotes m^−2^ s^−1^ and −4.4 eukaryotes m^−2^ s^−1^. Net fluxes of prokaryotes were negative in 35% of sampled locations, while the ocean was a net sink for eukaryotes at all stations sampled (Figure 6). The locations where the ocean acted as a net source of microorganisms to the atmosphere were mostly situated south of 60°N.

The estimated deposition rates indicated that the time necessary to deposit 50% of the suspended cells was on average 12 and 2 days for prokaryotes and eukaryotes, respectively (maximum 50 and 3.5 days). Modeling forward trajectories (Figure 1) for the time required to reduce the microbial load to 50% of the original revealed that half of the prokaryotes have the potential to be deposited after traveled distances between 1,000 and 300,000 km, with an average of 26,000 km, while 50% of the eukaryotes were still airborne after traveling from 300 to 3,000 km, with an average of 1,300 km.

The Discussion section, fourth paragraph, has been updated to reflect corrections to the calculated fluxes, residence times in the atmosphere, and traveled distances of prokaryotes and eukaryotes. The paragraph should read:

Estimations of airborne microbial abundance over the open ocean are needed to resolve the biology of the atmosphere, the Earth's biome where life is most diluted but that, as demonstrated for other similarly diluted constituents (e.g., nitrogen, pollutants, or gasses), plays a fundamental role in transport and connectivity across biomes (Uematsu et al., 1983; Krishnamurthy et al., 2010). Determining the concentration and loads of atmospheric microbes is an important step, and the estimates provided here suggest that microbes are diluted in the atmosphere more than 9 or 11 orders of magnitude relative to their concentration in seawater or soils (Whitman et al., 1998). This could lead to the conclusion that airborne bacteria are unimportant and can be neglected. Yet, the abundance of airborne microbes may be a misleading indicator of the importance of this compartment, as the atmosphere may play a major role in the dispersal of microbes, in the connectivity and the maintenance of diversity in the surface ocean or in regulating climatic processes through the role of airborne bacteria as nuclei of accretion for cloud and ice formation. Hence, while diluted relative to the marine or soil compartment, the estimated microbial load over the height of the boundary layer averaging 3.6 × 10^6^ prokaryotes m^−2^ and 7.6 × 10^5^ eukaryotes m^−2^ represents a formidable seed bank hovering over the North Atlantic Ocean. The net fluxes are relatively small (Figure 6) compared to those reported for land locations. Bacterial net flux measurements over a chaparral, oscillated over the day between −8 and 5 CFU m^2^ s^−1^ (Lighthart and Shaffer, 1994) while only upward fluxes up to 553 CFU m^2^ s^−1^ were reported for vegetated agricultural soils (Lindemann et al., 1982), if we consider that cultivable bacteria may be about 1% of the total bacterial load, our net bacterial fluxes could be up to four orders of magnitude lower. However, the microbes entering and leaving the surface ocean are not necessarily the same and thus, the calculated air-sea exchange of microbes shows that it is a highly dynamic process. Averages of 5 × 10^5^ prokaryotes and 5 × 10^2^ eukaryotes per square meter leaving the ocean into the atmosphere every day were calculated in this study, while an average of 4 × 10^5^ prokaryotes and eukaryotes per square meter enter the surface ocean from the atmosphere every day. These values evidence a rapid turnover of atmospheric microbes but also a high dispersal capacity since 50% of the microorganisms present in a given sample will still be airborne on average after 12 days in the case of prokaryotes and 2 days for eukaryotes. In other words, half of the microbes present in the original air sample will be deposited in a few days over large oceanic stretches of around thousands of kilometers long.

Finally, in Discussion section, final paragraph, it was stated that daily air-sea exchanges were in the order of millions of prokaryotes and thousands of unicellular eukaryotes. This has been corrected to hundreds of thousands. The corrected paragraph appears below:

In conclusion, we found atmospheric microbial abundances in the boundary layer over the North Atlantic Ocean ranging from 10^3^ to 10^4^ prokaryotes m^−3^ and from 10^2^ to 10^4^ eukaryotes m^−3^, but supporting daily air-sea exchanges in the order of hundreds of thousands of prokaryotes and unicellular eukaryotes per square meter of oceanic surface. This limited dataset provides a first snapshot of the microbial abundances and fluxes over the North Atlantic Ocean during our cruise. Additional efforts are needed to assess the temporal variability and the magnitude of these processes in this and other regions of the ocean. Calculations based on current parameterizations are crude and should be considered as order-of-magnitude estimates. Nevertheless, our data point to a rapid exchange of microbes between the atmosphere and the surface ocean, which is not apparent from abundance data only. This rapid flux could be of major importance for the dispersal of marine microbes and for the maintenance of local diversity over the global ocean.

## Conflict of interest statement

The authors declare that the research was conducted in the absence of any commercial or financial relationships that could be construed as a potential conflict of interest.
